# WebQUAST: online evaluation of genome assemblies

**DOI:** 10.1093/nar/gkad406

**Published:** 2023-05-17

**Authors:** Alla Mikheenko, Vladislav Saveliev, Pascal Hirsch, Alexey Gurevich

**Affiliations:** Department of Neuromuscular Diseases, UCL Queen Square Institute of Neurology, University College London, London WC1E 6BT, UK; Centre for Population Genomics, Garvan Institute of Medical Research and UNSW Sydney, Sydney, New South Wales 2010, Australia; Centre for Population Genomics, Murdoch Children’s Research Institute, Melbourne, Victoria 3052, Australia; Chair for Clinical Bioinformatics, Saarland University, Saarbrücken 66123, Germany; Helmholtz Institute for Pharmaceutical Research Saarland (HIPS), Helmholtz Centre for Infection Research, Saarbrücken 66123, Germany; Department of Computer Science, Saarland University, Saarbrücken 66123, Germany

## Abstract

Selecting proper genome assembly is key for downstream analysis in genomics studies. However, the availability of many genome assembly tools and the huge variety of their running parameters challenge this task. The existing online evaluation tools are limited to specific taxa or provide just a one-sided view on the assembly quality. We present WebQUAST, a web server for multifaceted quality assessment and comparison of genome assemblies based on the state-of-the-art QUAST tool. The server is freely available at https://www.ccb.uni-saarland.de/quast/. WebQUAST can handle an unlimited number of genome assemblies and evaluate them against a user-provided or pre-loaded reference genome or in a completely reference-free fashion. We demonstrate key WebQUAST features in three common evaluation scenarios: assembly of an unknown species, a model organism, and a close variant of it.

## Graphical Abstract

**Graphical Abstract:**
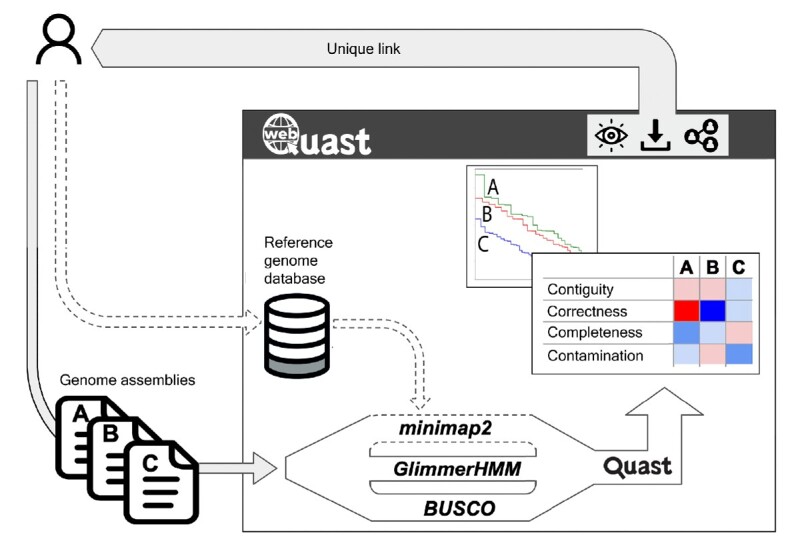

## INTRODUCTION

Despite the ongoing long-read sequencing revolution, it is still impossible to read entire chromosomes for most species in a single run (1). Researchers use the so-called genome assembly software that combines the sequencing reads into longer genome fragments commonly referred to as contigs. Dozens of genome assemblers exist nowadays (2). These tools rely on different heuristics that greatly vary their output. Moreover, even different settings of the same tool may result in substantially diverging assemblies. The quality assessment and comparison of multiple genome assemblies are of utmost importance since the assembly choice greatly affects the downstream analysis (3).

The existing assembly evaluation tools comprise two major categories. The reference-based tools, such as GAGE (4), use gold-standard reference genomes to evaluate assemblies on model datasets. The reference-free methods either rely on read mapping back to assemblies to check their consistency with the input data and detect assembly errors, such as REAPR (5) and Inspector (6), or look for conservative genes to estimate the assembly completeness, such as BUSCO (7,8) and CEGMA (9). Previously, we developed QUAST, an ensemble method that incorporated the best software from both categories, enhanced them with in-house quality metrics and plots, and became the state-of-the-art quality assessment tool for genome assemblies (10,11). However, QUAST intrinsically inherited the limitations of the embedded tools which are available only for a few platforms (usually Linux) and have a command-line interface making them hardly suitable for researchers with a limited computational background.

Here, we present WebQUAST, a web server complementing QUAST with a user-friendly graphical interface and providing its functionality on any platform. In contrast to a few existing genome assembly evaluation web tools, WebQUAST is not restricted to specific taxa as gEVAL (12) and GenomeQC (13), performs versatile assembly evaluation rather than only completeness estimation as gVolante (14), and supports an unlimited number of assemblies on input. The WebQUAST evaluation reports can be browsed online, downloaded locally, and shared privately with colleagues. We show WebQUAST performance using a sample dataset of four *E. coli* assemblies.

## MATERIALS AND METHODS

### Web server overview

#### Workflow

A user uploads genome assemblies in the FASTA format (gzipped files are supported), configures the evaluation parameters, such as the minimal contig length cut-off and the organism type (eukaryote or prokaryote), and optionally selects a reference genome. The user might choose it from the list of pre-loaded genomes or upload a custom FASTA file that will be stored privately and can be reused later. Once the user clicks on the Evaluate button, WebQUAST transfers the input data to the QUAST processing engine.

If a reference genome is provided, the assemblies are aligned against it using minimap2 (15). If the BUSCO checkbox is selected, the assemblies are screened for single-copy orthologues from the corresponding BUSCO database (8). If the gene finding is requested, the assemblies are processed with the GlimmerHMM gene prediction software (16). QUAST combines the outputs of all employed modules to compute numeric quality metrics, create assessment plots and Icarus viewers (17), and generate a single evaluation report. WebQUAST assigns the report a unique web link and renders it for the user. The link enables browsing the results online and sharing them. The user can download the full standalone report to store it permanently. The standalone report also provides additional insights into the analysis, such as the running commands of the embedded tools or the list of identified misassemblies in the GFF format.

#### Software implementation

The server is built on top of the Python web framework Django. MySQL instance is used to record users, sessions, and analysis requests. To support long-running analysis, the requests are processed and added into an asynchronous task queue Celery. A queued job represents a simple script that calls the command-line QUAST tool, which allows us to keep the main codebase agnostic to the web implementation. The front-end component is based on the jQuery framework.

### Sample data preparation

To demonstrate WebQUAST performance, we generated sample assemblies of a well-studied short-read *Escherichia coli* K-12 MG1655 dataset (SRA accession: ERR008613). The choice of a genome assembler might be influenced by many factors and one popular, yet often suboptimal, strategy is to choose among the most-cited methods (18). We mimicked this behavior by collecting information on short-read genome assemblers (Table 1) and selecting the five most-cited tools. We further excluded SOAPdenovo (19) since the authors discontinued it and recommended using MEGAHIT (20), which was already shortlisted.

**Table 1.** The most-cited short-read genome assemblers

Assembler	Latest release	Num citations		Key publications
	version (year)	total	yearly	with years
SPAdes	3.15.5 (2022)	18833	1847	2020 (21), 2012 (22)
Velvet	1.2.10 (2014)	10633	709	2008 (23)
SOAPdenovo	242 (2018)	7410	630	2012 (19), 2010 (24)
MEGAHIT	1.2.9 (2019)	4519	581	2016 (20), 2015 (25)
ABySS	2.3.5 (2022)	4445	366	2017 (26), 2009 (27)
IDBA	1.1.3 (2016)	2979	266	2012 (28), 2010 (29)
ALLPATHS	52488 (2016)	2868	210	2011 (30), 2008 (31)
MaSuRCA	4.1.0 (2023)	1434	164	2017 (32), 2013 (33)
Ray	2.3.1 (2014)	1232	103	2012 (34), 2010 (35)
SGA	0.10.15 (2016)	909	83	2012 (36)

Version numbers and dates of the latest release were determined from the GitHub repositories of the tools. *Num citations* stands for the number of citations according to Google.Scholar as of 28.03.2022, *yearly* average is the total number of citations divided by the sum of full years past since the publications. At most two key publications per tool are included; if there were more than two publications, we relied on the citation recommendations on the tool webpage (usually the first and the last publication).

Some of the selected assemblers do not include a read error correction module, so we cleaned the raw sequencing data beforehand to make the comparison fair. We checked the reads with FastQC and trimmed low-quality ends with Trimmomatic (37). All assemblers but ABySS were run with default parameters or based on the recommendations in the documentation wherever available. We used the GAGE-B recipe (38) for ABySS since its default assembly was of very poor quality. All tools were installed via Bioconda (39), the installation and running commands are in the Supplementary Material.

## RESULTS

Here we illustrate three typical WebQUAST usage scenarios. In each case, we evaluated the same four assemblies of the *E. coli* K-12 MG1655 dataset but selected the reference genome differently. We assumed the reference was unknown in Case 1, exactly matched the dataset in Case 2, and was closely related to the dataset in Case 3.

### Use Case 1: reference-free evaluation

**Figure 1.**
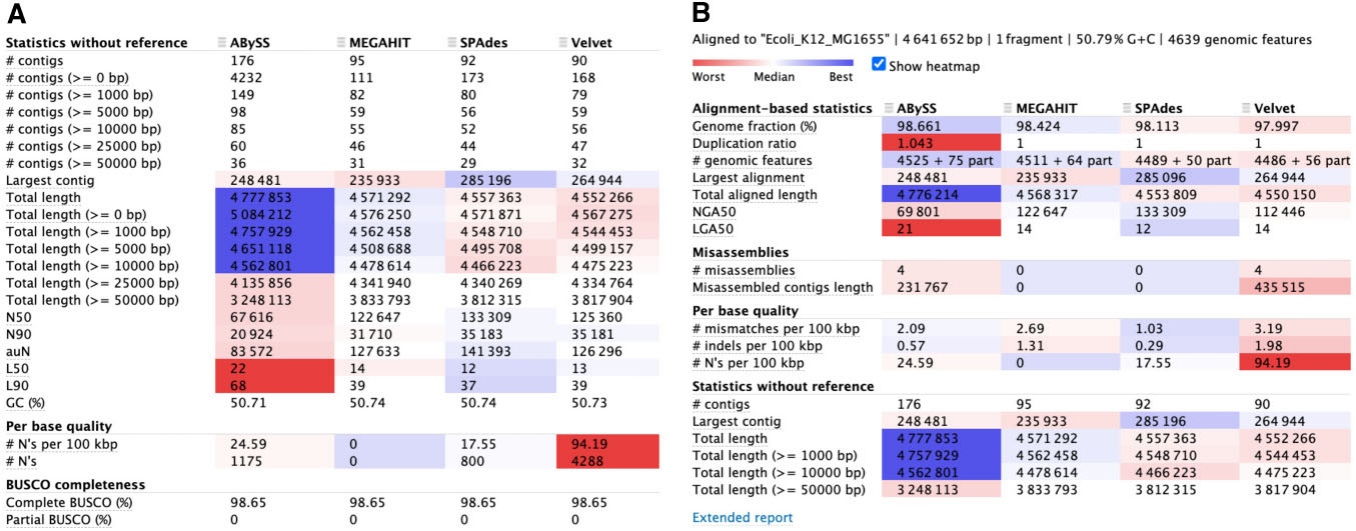
WebQUAST text reports for *E. coli* assemblies in the (**A**) reference-free and (**B**) reference-based evaluation mode. Unless otherwise noted, all statistics are based on contigs of size ≥ 500 bp (the default cut-off). Heatmap highlights the best value in each row which could be the largest or the smallest number depending on the quality metric. Heatmap is not used for *# contigs* and *GC (%)* due to the ambiguity of these metrics trends.

When a reference genome is unavailable, WebQUAST computes 30 quality metrics and draws three assessment plots that mainly address the contiguity and completeness of the provided assemblies (Figure 1A, Supplementary Figure S1). The heatmaps help to detect the best-performing tools in each category.

Figure 1A shows that there is no single winner in all metrics. Compared to three other methods, ABySS produced the largest (4.8 Mb versus 4.6 Mb) but also the most fragmented assembly (176 contigs versus 90–95 for Velvet, SPAdes and MEGAHIT). SPAdes assembled larger contigs on average (the best N50, N90 and auN, the area under the Nx curve, values with Velvet and MEGAHIT being close runner-ups) and has the largest contig overall (285 versus 265, 248 and 236 kb for Velvet, ABySS and MEGAHIT). The MEGAHIT assembly does not contain uncalled bases (‘N’) while Velvet has the most of them (94 per 100 kb). All four assemblies are equally complete in terms of fully assembled representative bacterial single-copy orthologs (98.7% of the BUSCO genes). The average G + C content of all assemblies (50.7%) perfectly matches the expected range for *E. coli* (50.4–50.8% (40)) indicating the likely absence of contaminants in the dataset. This hypothesis is further supported by the GC plot (Supplementary Figure S1D), though we cannot exclude a presence of an organism with similar G + C content.

### Use Case 2: reference-based evaluation

**Figure 2.**
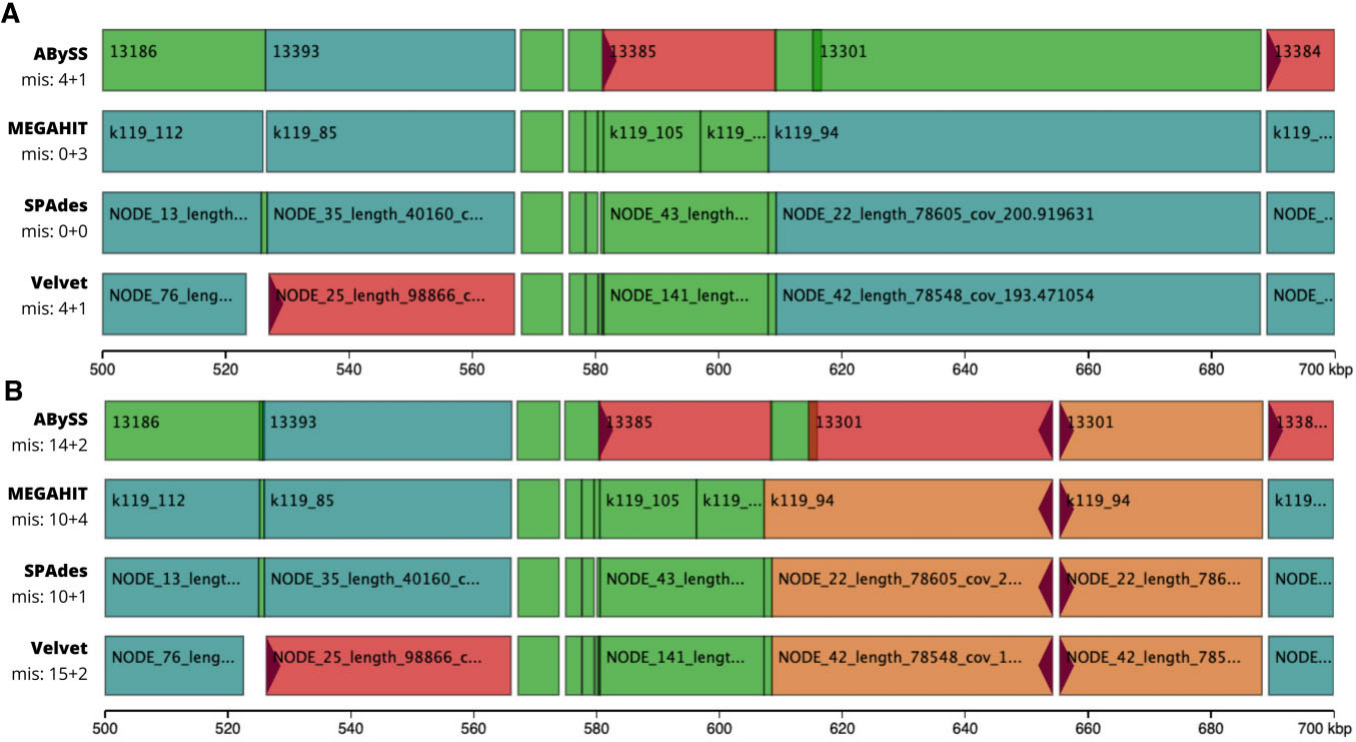
Icarus viewers for *E. coli* assemblies aligned against (**A**) the reference genome matching the dataset and (**B**) a close reference. The reference regions between 0.5 Mb and 0.7 Mb are shown. *mis: X + Y* stands for the total number of extensive (*X*) and local (*Y*) misassemblies per assembly. Correctly assembled contigs are colored green and aquamarine (if longer than 10 kb and similar in at least three assemblies), and fragments of misassembled contigs are colored pink and orange (if similar in at least three assemblies). Red triangles designate the sides of alignment breakpoints for misassembled contigs. Contig names are shown for contigs of sufficient size.

A reference genome enables accurate and versatile evaluation by WebQUAST in all four quality categories: contiguity, correctness, completeness, and contamination. In this mode, the tool reports >60 quality metrics accompanied by eight assessment plots and two Icarus viewers (Figure 1B, Figure 2A, Supplementary Figures S2–S4). By default, WebQUAST displays only 18 key metrics and hides the rest behind the Extended report button (Figure 1B).

As in Use Case 1, there is no undisputed best assembly in Figure 1B. However, we can now investigate some quality categories in more detail. The increased Duplication ratio for ABySS (1.04 versus 1.00 for the rest assemblers) indicates that this method assembled many genomic regions more than once. Still, ABySS assembled the highest percentage of the genome (98.7 versus 98.0-98.4% for Velvet, SPAdes and MEGAHIT) but its leadership is not as evident as it appeared when we compared the total assembly lengths. SPAdes and ABySS have the best per-base quality with SPAdes being twice better as the runner-up (1.0 vs 2.1 mismatches and 0.3 versus 0.6 indels per 100 kb). MEGAHIT and SPAdes made no large assembly errors, while Velvet and ABySS have four misassemblies each. Though, the largest contigs in all four assemblies are error-free since their lengths exactly match the largest alignments. The Icarus viewer can be used for deep inspection of the misassembly locations (Figure 2, Supplementary Figure S4).

### Use case 3: evaluation based on a close reference

The true reference genome is rarely known in real studies but a close reference could often be available. Here we used W3110, another *E. coli* K-12 substrain, as an example of a close reference (Figure 2B, Supplementary Figures S5–S7). Naturally, the absolute values of many alignment-based metrics, such as lengths of misassembled and unaligned contigs, substantially deteriorated due to the actual differences between the sequenced organism and the provided reference genome. However, they are still useful for determining the best assembly among available options.

Figure 2 highlights the substantially increased number of misassemblies compared to the evaluation based on the true reference genome (49 versus 8 extensive misassemblies in total). However, a closer look at the misassembly locations, suggests that almost all of them are the same in all assemblies which likely means they are true structural variations rather than assembly errors and can be ignored for evaluation purposes (Figure 2B and Supplementary Figure S7). Though, we cannot exclude the possibility that several assemblers made the same error in a complex genomic region, especially if we compare tools inspired by the same computational approach such as the de Bruijn graph-based assembly (41).

## CONCLUSION

Selecting the best – or, more precisely, the most suitable – genome assembly is crucial for downstream analysis. While many post-processing steps, such as structural and functional annotation (42) or genome mining (43), have been available online for years, the assembly validation step is still mainly done with the Linux-based command-line tools. Here, we presented WebQUAST, a web server for genome assembly evaluation, that greatly facilitates this task for users with any operating system and computational background and helps them to make an informed choice. Since our tool is suitable for any organism and sequencing technology, we expect it would benefit the broad genomics community. Furthermore, WebQUAST is already incorporated in several bioinformatics massive online open courses (MOOCs), so we hope it would also help to educate the future generation of researchers.

## DATA AVAILABILITY

WebQUAST is freely available at https://www.ccb.uni-saarland.de/quast/. The source code for the server is at https://github.com/ablab/quast-website and for the core QUAST tool is at https://github.com/ablab/quast. The sequencing data for *E. coli* K-12 MG1655 dataset is available from the National Center for Biotechnology Information (NCBI) Sequence Read Archive under accession number ERR008613. The *E. coli* strain K-12 reference genomes and gene annotations are available from NCBI under accession numbers NC_000913.3 and AP009048.1 for substrains MG1655 and W3110, respectively. The ABySS, MEGAHIT, SPAdes, and Velvet assemblies generated in this study and their interactive evaluation reports are available from the WebQUAST front page and in Zenodo at https://doi.org/10.5281/zenodo.7863703.

## Supplementary Material

gkad406_Supplemental_File

## References

[1] Van Dijk E.L. , Jaszczyszyn Y., Naquin D., Thermes C. The third revolution in sequencing technology. Trends Genet. 2018; 34:666–681. 29941292 10.1016/j.tig.2018.05.008

[2] Sohn J.-i. , Nam J.-W. The present and future of de novo whole-genome assembly. Brief. Bioinform. 2018; 19:23–40. 27742661 10.1093/bib/bbw096

[3] Lloret-Villas A. , Bhati M., Kadri N.K., Fries R., Pausch H. Investigating the impact of reference assembly choice on genomic analyses in a cattle breed. BMC Genomics. 2021; 22:1–17. 34011274 10.1186/s12864-021-07554-w PMC8132449

[4] Salzberg S.L. , Phillippy A.M., Zimin A., Puiu D., Magoc T., Koren S., Treangen T.J., Schatz M.C., Delcher A.L., Roberts M. et al. GAGE: a critical evaluation of genome assemblies and assembly algorithms. Genome Res. 2012; 22:557–567. 22147368 10.1101/gr.131383.111 PMC3290791

[5] Hunt M. , Kikuchi T., Sanders M., Newbold C., Berriman M., Otto T.D. REAPR: a universal tool for genome assembly evaluation. Genome Biol. 2013; 14:1–10. 10.1186/gb-2013-14-5-r47 PMC3798757 23710727

[6] Chen Y. , Zhang Y., Wang A.Y., Gao M., Chong Z. Accurate long-read de novo assembly evaluation with Inspector. Genome Biol. 2021; 22:1–21. 34775997 10.1186/s13059-021-02527-4 PMC8590762

[7] Simão F.A. , Waterhouse R.M., Ioannidis P., Kriventseva E.V., Zdobnov E.M. BUSCO: assessing genome assembly and annotation completeness with single-copy orthologs. Bioinformatics. 2015; 31:3210–3212. 26059717 10.1093/bioinformatics/btv351

[8] Seppey M. , Manni M., Zdobnov E.M. BUSCO: assessing genome assembly and annotation completeness. Gene Prediction: Methods and Protocols. 2019; 1962:227–245. 10.1007/978-1-4939-9173-0_14 31020564

[9] Parra G. , Bradnam K., Korf I. CEGMA: a pipeline to accurately annotate core genes in eukaryotic genomes. Bioinformatics. 2007; 23:1061–1067. 17332020 10.1093/bioinformatics/btm071

[10] Gurevich A. , Saveliev V., Vyahhi N., Tesler G. QUAST: quality assessment tool for genome assemblies. Bioinformatics. 2013; 29:1072–1075. 23422339 10.1093/bioinformatics/btt086 PMC3624806

[11] Mikheenko A. , Prjibelski A., Saveliev V., Antipov D., Gurevich A. Versatile genome assembly evaluation with QUAST-LG. Bioinformatics. 2018; 34:i142–i150. 29949969 10.1093/bioinformatics/bty266 PMC6022658

[12] Chow W. , Brugger K., Caccamo M., Sealy I., Torrance J., Howe K. gEVAL—a web-based browser for evaluating genome assemblies. Bioinformatics. 2016; 32:2508–2510. 27153597 10.1093/bioinformatics/btw159 PMC4978925

[13] Manchanda N. , Portwood J.L., Woodhouse M.R., Seetharam A.S., Lawrence-Dill C.J., Andorf C.M., Hufford M.B. GenomeQC: a quality assessment tool for genome assemblies and gene structure annotations. BMC Genomics. 2020; 21:1–9. 10.1186/s12864-020-6568-2 PMC7053122 32122303

[14] Nishimura O. , Hara Y., Kuraku S. gVolante for standardizing completeness assessment of genome and transcriptome assemblies. Bioinformatics. 2017; 33:3635–3637. 29036533 10.1093/bioinformatics/btx445 PMC5870689

[15] Li H. Minimap2: pairwise alignment for nucleotide sequences. Bioinformatics. 2018; 34:3094–3100. 29750242 10.1093/bioinformatics/bty191 PMC6137996

[16] Majoros W.H. , Pertea M., Salzberg S.L. TigrScan and GlimmerHMM: two open source ab initio eukaryotic gene-finders. Bioinformatics. 2004; 20:2878–2879. 15145805 10.1093/bioinformatics/bth315

[17] Mikheenko A. , Valin G., Prjibelski A., Saveliev V., Gurevich A. Icarus: visualizer for de novo assembly evaluation. Bioinformatics. 2016; 32:3321–3323. 27378299 10.1093/bioinformatics/btw379

[18] Gardner P.P. , Paterson J.M., McGimpsey S., Ashari-Ghomi F., Umu S.U., Pawlik A., Gavryushkin A., Black M.A. Sustained software development, not number of citations or journal choice, is indicative of accurate bioinformatic software. Genome Biol. 2022; 23:1–13. 35172880 10.1186/s13059-022-02625-x PMC8851831

[19] Luo R. , Liu B., Xie Y., Li Z., Huang W., Yuan J., He G., Chen Y., Pan Q., Liu Y. et al. SOAPdenovo2: an empirically improved memory-efficient short-read de novo assembler. Gigascience. 2012; 1:18. 23587118 10.1186/2047-217X-1-18 PMC3626529

[20] Li D. , Luo R., Liu C.-M., Leung C.-M., Ting H.-F., Sadakane K., Yamashita H., Lam T.-W. MEGAHIT v1. 0: a fast and scalable metagenome assembler driven by advanced methodologies and community practices. Methods. 2016; 102:3–11. 27012178 10.1016/j.ymeth.2016.02.020

[21] Prjibelski A. , Antipov D., Meleshko D., Lapidus A., Korobeynikov A. Using SPAdes de novo assembler. Curr. Prot. Bioinform. 2020; 70:e102. 10.1002/cpbi.102 32559359

[22] Bankevich A. , Nurk S., Antipov D., Gurevich A.A., Dvorkin M., Kulikov A.S., Lesin V.M., Nikolenko S.I., Pham S., Prjibelski A.D. et al. SPAdes: a new genome assembly algorithm and its applications to single-cell sequencing. J. Comput. Biol. 2012; 19:455–477. 22506599 10.1089/cmb.2012.0021 PMC3342519

[23] Zerbino D.R. , Birney E. Velvet: algorithms for de novo short read assembly using de Bruijn graphs. Genome Res. 2008; 18:821–829. 18349386 10.1101/gr.074492.107 PMC2336801

[24] Li R. , Zhu H., Ruan J., Qian W., Fang X., Shi Z., Li Y., Li S., Shan G., Kristiansen K. et al. De novo assembly of human genomes with massively parallel short read sequencing. Genome Res. 2010; 20:265–272. 20019144 10.1101/gr.097261.109 PMC2813482

[25] Li D. , Liu C.-M., Luo R., Sadakane K., Lam T.-W. MEGAHIT: an ultra-fast single-node solution for large and complex metagenomics assembly via succinct de Bruijn graph. Bioinformatics. 2015; 31:1674–1676. 25609793 10.1093/bioinformatics/btv033

[26] Jackman S.D. , Vandervalk B.P., Mohamadi H., Chu J., Yeo S., Hammond S.A., Jahesh G., Khan H., Coombe L., Warren R.L. et al. ABySS 2.0: resource-efficient assembly of large genomes using a Bloom filter. Genome Res. 2017; 27:768–777. 28232478 10.1101/gr.214346.116 PMC5411771

[27] Simpson J.T. , Wong K., Jackman S.D., Schein J.E., Jones S.J., Birol I. ABySS: a parallel assembler for short read sequence data. Genome Res. 2009; 19:1117–1123. 19251739 10.1101/gr.089532.108 PMC2694472

[28] Peng Y. , Leung H.C., Yiu S.-M., Chin F.Y. IDBA-UD: a de novo assembler for single-cell and metagenomic sequencing data with highly uneven depth. Bioinformatics. 2012; 28:1420–1428. 22495754 10.1093/bioinformatics/bts174

[29] Peng Y. , Leung H.C., Yiu S.-M., Chin F.Y. IDBA–a practical iterative de Bruijn graph de novo assembler. In Research in Computational Molecular Biology: 14th Annual International Conference, RECOMB 2010, Lisbon, Portugal, April 25-28, 2010. Proceedings 14. 2010; Springer 426–440.

[30] Gnerre S. , MacCallum I., Przybylski D., Ribeiro F.J., Burton J.N., Walker B.J., Sharpe T., Hall G., Shea T.P., Sykes S. et al. High-quality draft assemblies of mammalian genomes from massively parallel sequence data. Proc. Natl. Acad. Sci. U.S.A. 2011; 108:1513–1518. 21187386 10.1073/pnas.1017351108 PMC3029755

[31] Butler J. , MacCallum I., Kleber M., Shlyakhter I.A., Belmonte M.K., Lander E.S., Nusbaum C., Jaffe D.B. ALLPATHS: de novo assembly of whole-genome shotgun microreads. Genome Res. 2008; 18:810–820. 18340039 10.1101/gr.7337908 PMC2336810

[32] Zimin A.V. , Puiu D., Luo M.-C., Zhu T., Koren S., Marçais G., Yorke J.A., Dvořák J., Salzberg S.L. Hybrid assembly of the large and highly repetitive genome of Aegilops tauschii, a progenitor of bread wheat, with the MaSuRCA mega-reads algorithm. Genome Res. 2017; 27:787–792. 28130360 10.1101/gr.213405.116 PMC5411773

[33] Zimin A.V. , Marçais G., Puiu D., Roberts M., Salzberg S.L., Yorke J.A. The MaSuRCA genome assembler. Bioinformatics. 2013; 29:2669–2677. 23990416 10.1093/bioinformatics/btt476 PMC3799473

[34] Boisvert S. , Raymond F., Godzaridis É., Laviolette F., Corbeil J. Ray Meta: scalable de novo metagenome assembly and profiling. Genome Biol. 2012; 13:1–13. 10.1186/gb-2012-13-12-r122 PMC4056372 23259615

[35] Boisvert S. , Laviolette F., Corbeil J. Ray: simultaneous assembly of reads from a mix of high-throughput sequencing technologies. J. Comput. Biol. 2010; 17:1519–1533. 20958248 10.1089/cmb.2009.0238 PMC3119603

[36] Simpson J.T. , Durbin R. Efficient de novo assembly of large genomes using compressed data structures. Genome Res. 2012; 22:549–556. 22156294 10.1101/gr.126953.111 PMC3290790

[37] Bolger A.M. , Lohse M., Usadel B. Trimmomatic: a flexible trimmer for Illumina sequence data. Bioinformatics. 2014; 30:2114–2120. 24695404 10.1093/bioinformatics/btu170 PMC4103590

[38] Magoc T. , Pabinger S., Canzar S., Liu X., Su Q., Puiu D., Tallon L.J., Salzberg S.L. GAGE-B: an evaluation of genome assemblers for bacterial organisms. Bioinformatics. 2013; 29:1718–1725. 23665771 10.1093/bioinformatics/btt273 PMC3702249

[39] Grüning B. , Dale R., Sjödin A., Chapman B.A., Rowe J., Tomkins-Tinch C.H., Valieris R., Köster J., Team B. Bioconda: sustainable and comprehensive software distribution for the life sciences. Nat. Methods. 2018; 15:475–476. 29967506 10.1038/s41592-018-0046-7 PMC11070151

[40] Mann S. , Chen Y.-P.P. Bacterial genomic G+C composition-eliciting environmental adaptation. Genomics. 2010; 95:7–15. 19747541 10.1016/j.ygeno.2009.09.002

[41] Pevzner P.A. , Tang H., Waterman M.S. An Eulerian path approach to DNA fragment assembly. Proc. Natl. Acad. Sci. U.S.A. 2001; 98:9748–9753. 11504945 10.1073/pnas.171285098 PMC55524

[42] Humann J.L. , Lee T., Ficklin S., Main D. Structural and functional annotation of eukaryotic genomes with GenSAS. Gene Prediction: Methods Protoc. 2019; 1962:29–51. 10.1007/978-1-4939-9173-0_3 31020553

[43] Blin K. , Shaw S., Kloosterman A.M., Charlop-Powers Z., Van Wezel G.P., Medema M.H., Weber T. antiSMASH 6.0: improving cluster detection and comparison capabilities. Nucleic Acids Res. 2021; 49:W29–W35. 33978755 10.1093/nar/gkab335 PMC8262755

