# Supplementary material for: WebQUAST: online evaluation of genome assemblies
Source: Nucleic Acids Res. 2023 May 17;51(W1):W601–6. doi: 10.1093/nar/gkad406 (PMC10320133; doi:10.1093/nar/gkad406)
Supplement: gkad406_Supplemental_File [file gkad406_supplemental_file.pdf]

Supplementary Material for

WebQUAST: online evaluation of genome assemblies

Alla Mikheenko[1,†], Vladislav Saveliev[2,3,†], Pascal Hirsch[4], and Alexey Gurevich[5,6,*]

[1] Department of Neuromuscular Diseases, UCL Queen Square Institute of Neurology, University College London, London WC1E 6BT, UK
[2] Centre for Population Genomics, Garvan Institute of Medical Research and UNSW Sydney, Sydney, New South Wales 2010, Australia
[3] Centre for Population Genomics, Murdoch Children's Research Institute, Melbourne, Victoria 3052, Australia
[4] Chair for Clinical Bioinformatics, Saarland University, Saarbrücken 66123, Germany
[5] Helmholtz Institute for Pharmaceutical Research Saarland (HIPS), Helmholtz Centre for Infection Research, Saarbrücken 66123, Germany
[6] Department of Computer Science, Saarland University, Saarbrücken 66123, Germany

* To whom correspondence should be addressed. Email: alexey.gurevich@helmholtz-hips.de
† The authors wish it to be known that, in their opinion, the first two authors should be regarded as Joint First Authors.

Supplementary Figures

A

Worst Median Best

Show heatmap

Statistics without reference	ABySS	MEGAHIT	SPAdes	Velvet
# contigs	176	95	92	90
# contigs (>= 0 bp)	4232	111	173	168
# contigs (>= 1000 bp)	149	82	80	79
# contigs (>= 5000 bp)	98	59	56	59
# contigs (>= 10000 bp)	85	55	52	56
# contigs (>= 25000 bp)	60	46	44	47
# contigs (>= 50000 bp)	36	31	29	32
Largest contig	248 481	235 933	285 196	264 944
Total length	4 777 853	4 571 292	4 557 363	4 552 266
Total length (>= 0 bp)	5 084 212	4 576 250	4 571 871	4 567 275
Total length (>= 1000 bp)	4 757 929	4 562 458	4 548 710	4 544 453
Total length (>= 5000 bp)	4 651 118	4 508 688	4 495 708	4 499 157
Total length (>= 10000 bp)	4 562 801	4 478 614	4 466 223	4 475 223
Total length (>= 25000 bp)	4 135 856	4 341 940	4 340 269	4 334 764
Total length (>= 50000 bp)	3 248 113	3 833 793	3 812 315	3 817 904
N50	67 616	122 647	133 309	125 360
N90	20 924	31 710	35 183	35 181
auN	83 572	127 633	141 393	126 296
L50	22	14	12	13
L90	68	39	37	39
GC (%)	50.71	50.74	50.74	50.73
**Per base quality**				
# N's per 100 kbp	24.59	0	17.55	94.19
# N's	1175	0	800	4288
**BUSCO completeness**				
Complete BUSCO (%)	98.65	98.65	98.65	98.65
Partial BUSCO (%)	0	0	0	0
**Predicted genes**				
# predicted genes (unique)	3717	3595	3587	3576
# predicted genes (>= 0 bp)	3778 + 38 part	3586 + 9 part	3585 + 2 part	3568 + 8 part
# predicted genes (>= 300 bp)	3534 + 30 part	3374 + 7 part	3367 + 2 part	3357 + 7 part
# predicted genes (>= 1500 bp)	670 + 2 part	663 + 0 part	662 + 0 part	657 + 1 part
# predicted genes (>= 3000 bp)	84 + 0 part	80 + 0 part	85 + 0 part	79 + 0 part

B

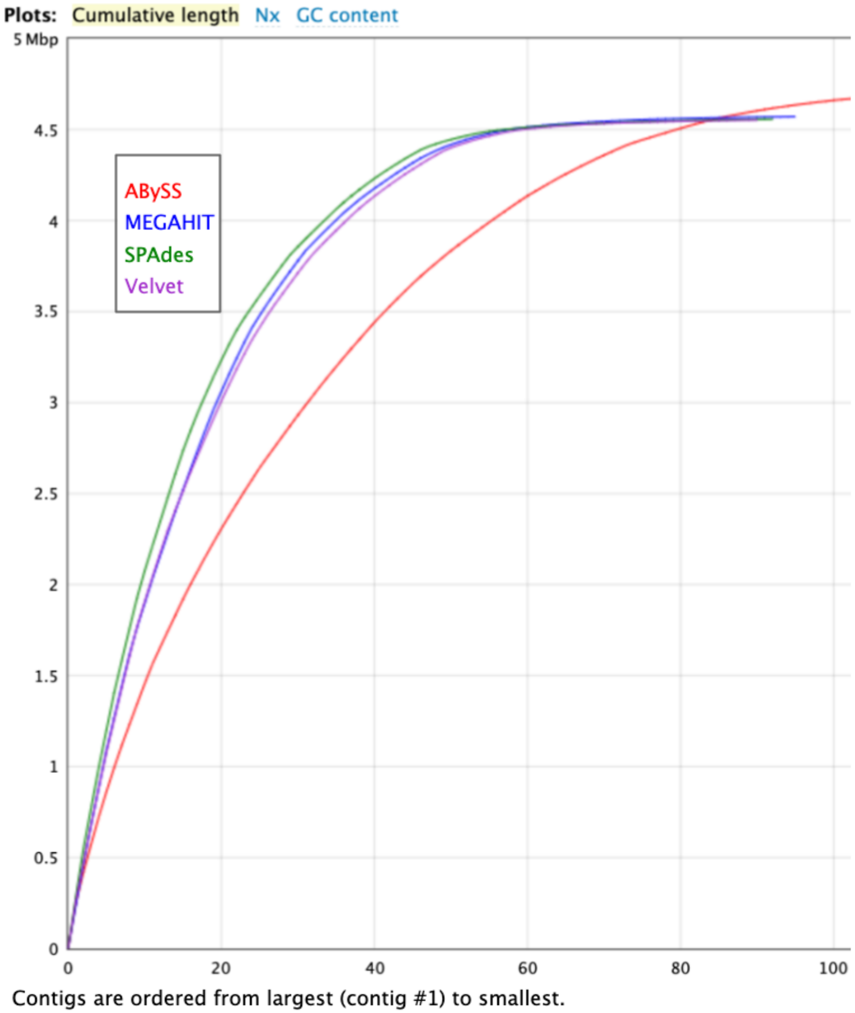

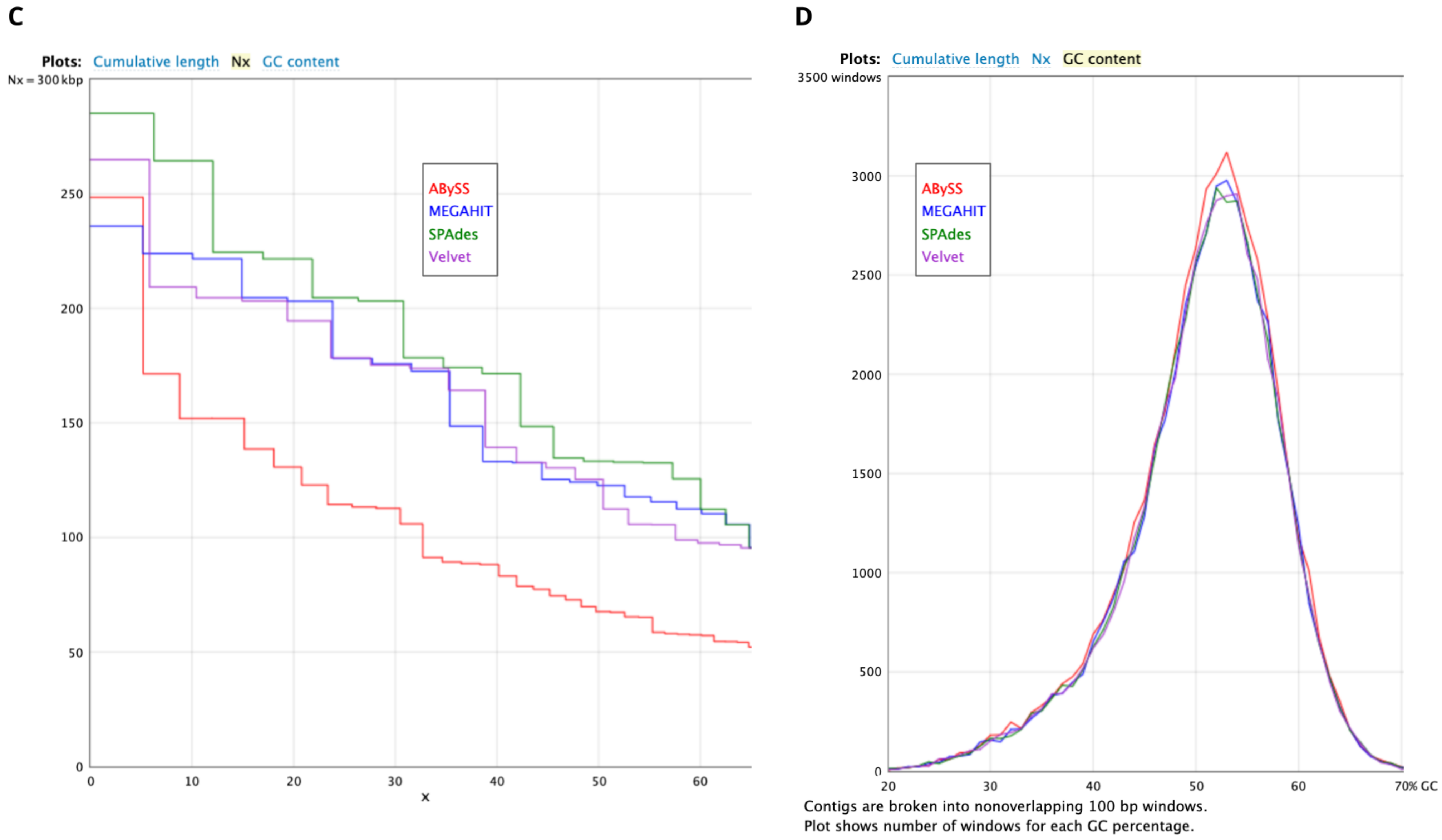

Supplementary Figure S1. Main WebQUAST report for the reference-free evaluation of *E. coli* assemblies (Use Case 1). (**A**) Numeric quality metrics. (**B**) Cumulative length plot. (**C**) Nx plot. (**D**) GC content plot. Unless otherwise noted, all statistics and plots are based on contigs of size ≥ 500 bp (the default cut-off). The full interactive report is available at https://www.ccb.uni-saarland.de/quast/sample_data_no_ref/report.html.

Aligned to "Ecoli_K12_MG1655" | 4 641 652 bp | 1 fragment | 50.79% G+C
4639 genomic features

Worst Median Best

Show heatmap

Alignment-based statistics	ABySS	MEGAHIT	SPAdes	Velvet
Genome fraction (%)	98.661	98.424	98.113	97.997
Duplication ratio	1.043	1	1	1
# genomic features	4525 + 75 part	4511 + 64 part	4489 + 50 part	4486 + 56 part
Largest alignment	248 481	235 933	285 096	264 944
Total aligned length	4 776 214	4 568 317	4 553 809	4 550 150
NG50	69 801	122 647	133 309	112 446
NG90	24 486	29 140	31 582	30 841
auNG	86 024	125 698	138 825	123 864
NA50	67 616	122 647	133 309	112 446
NA90	19 898	31 710	35 183	33 429
auNA	81 793	127 607	141 346	121 261
NGA50	69 801	122 647	133 309	112 446
NGA90	24 202	29 140	31 582	30 841
auNGA	84 193	125 672	138 780	118 926
LG50	21	14	12	14
LG90	62	41	39	42
LA50	22	14	12	14
LA90	69	39	37	42
LGA50	21	14	12	14
LGA90	64	41	39	44
**Misassemblies**				
# misassemblies	4	0	0	4
# relocations	4	0	0	4
# translocations	0	0	0	0
# inversions	0	0	0	0
# misassembled contigs	4	0	0	4
Misassembled contigs length	231 767	0	0	435 515
# local misassemblies	1	3	0	1
# scaffold gap ext. mis.	0	0	0	0
# scaffold gap loc. mis.	11	0	4	4
# unaligned mis. contigs	0	0	0	0
**Unaligned**				
# fully unaligned contigs	0	3	4	0
Fully unaligned length	0	2238	2936	0
# partially unaligned contigs	0	0	0	0
Partially unaligned length	0	0	0	0

(continued)

Worst Median Best

Show heatmap

Per base quality	ABySS	MEGAHIT	SPAdes	Velvet
# mismatches per 100 kbp	2.09	2.69	1.03	3.19
# mismatches	100	123	47	145
# indels per 100 kbp	0.57	1.31	0.29	1.98
# indels	27	60	13	90
# indels (<= 5 bp)	9	34	6	25
# indels (> 5 bp)	18	26	7	65
Indels length	1757	3403	1092	4620
# N's per 100 kbp	24.59	0	17.55	94.19
# N's	1175	0	800	4288
**Statistics without reference**				
# contigs	176	95	92	90
# contigs (>= 0 bp)	4232	111	173	168
# contigs (>= 1000 bp)	149	82	80	79
# contigs (>= 5000 bp)	98	59	56	59
# contigs (>= 10000 bp)	85	55	52	56
# contigs (>= 25000 bp)	60	46	44	47
# contigs (>= 50000 bp)	36	31	29	32
Largest contig	248 481	235 933	285 196	264 944
Total length	4 777 853	4 571 292	4 557 363	4 552 266
Total length (>= 0 bp)	5 084 212	4 576 250	4 571 871	4 567 275
Total length (>= 1000 bp)	4 757 929	4 562 458	4 548 710	4 544 453
Total length (>= 5000 bp)	4 651 118	4 508 688	4 495 708	4 499 157
Total length (>= 10000 bp)	4 562 801	4 478 614	4 466 223	4 475 223
Total length (>= 25000 bp)	4 135 856	4 341 940	4 340 269	4 334 764
Total length (>= 50000 bp)	3 248 113	3 833 793	3 812 315	3 817 904
N50	67 616	122 647	133 309	125 360
N90	20 924	31 710	35 183	35 181
auN	83 572	127 633	141 393	126 296
L50	22	14	12	13
L90	68	39	37	39
GC (%)	50.71	50.74	50.74	50.73
**Similarity statistics**				
# similar correct contigs	16	33	34	32
# similar misassembled blocks	0	0	0	0

Short report

Supplementary Figure S2. Extended WebQUAST text report for *E. coli* assemblies evaluated against the corresponding reference genome, *E.coli* str. K-12 substr. MG1655 (Use Case 2). Unless otherwise noted, all statistics are based on contigs of size ≥ 500 bp (the default cut-off). The full interactive report is available at https://www.ccb.uni-saarland.de/quast/sample_data_true_ref/report.html.

A

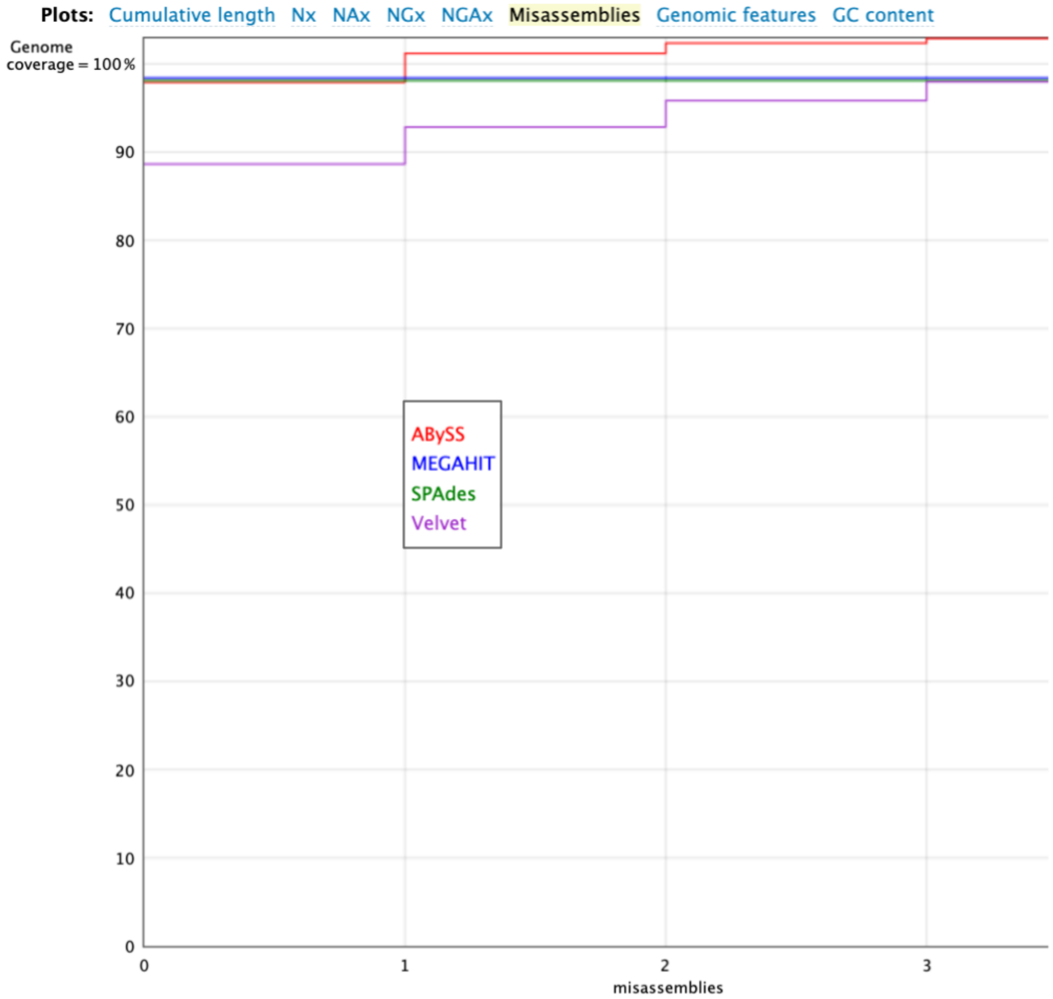

FRCurve: Y is the total number of aligned bases divided by the reference length, in the contigs having the total number of misassemblies at most X.

B

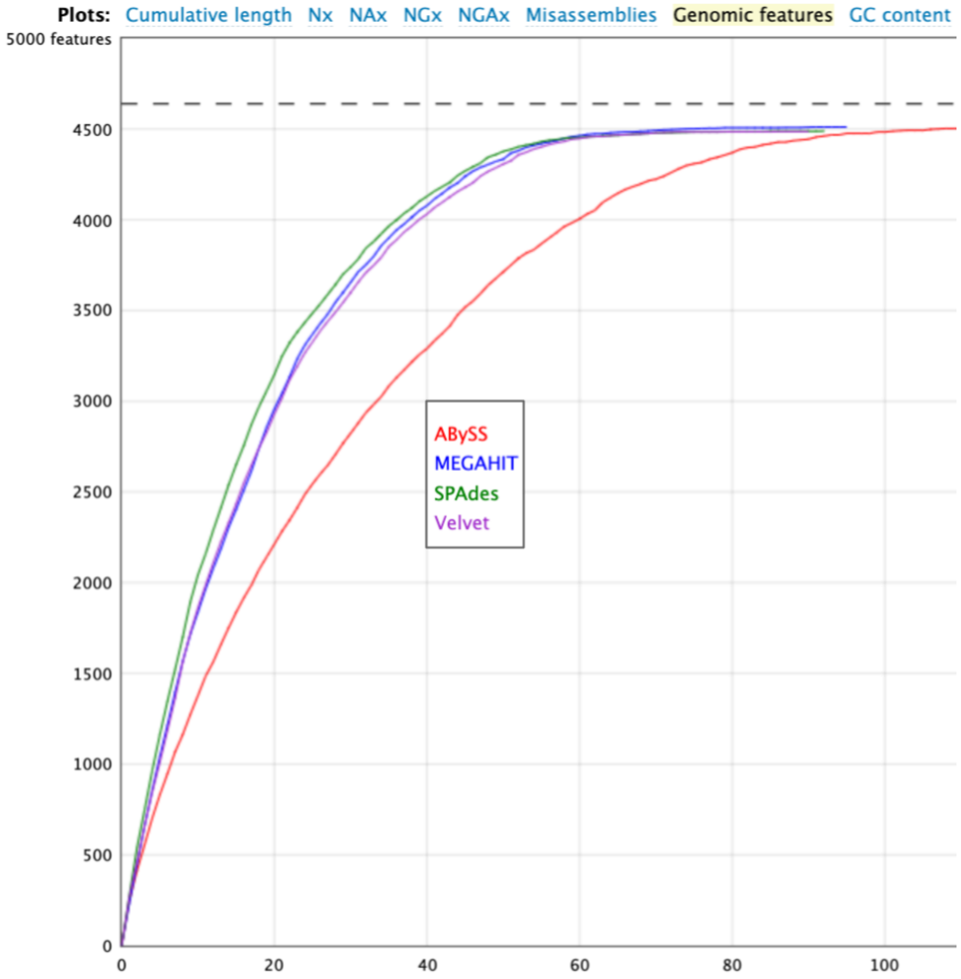

Contigs are ordered from largest (contig #1) to smallest.

Supplementary Figure S3. WebQUAST plots for *E. coli* assemblies evaluated against the corresponding reference genome, *E.coli* str. K-12 substr. MG1655 (Use Case 2). (**A**) Misassemblies feature-response curve. (**B**) Cumulative number of genes plot. Both plots are based on contigs of size ≥ 500 bp (the default cut-off). The full interactive report is available at https://www.ccb.uni-saarland.de/quast/sample_data_true_ref/report.html.

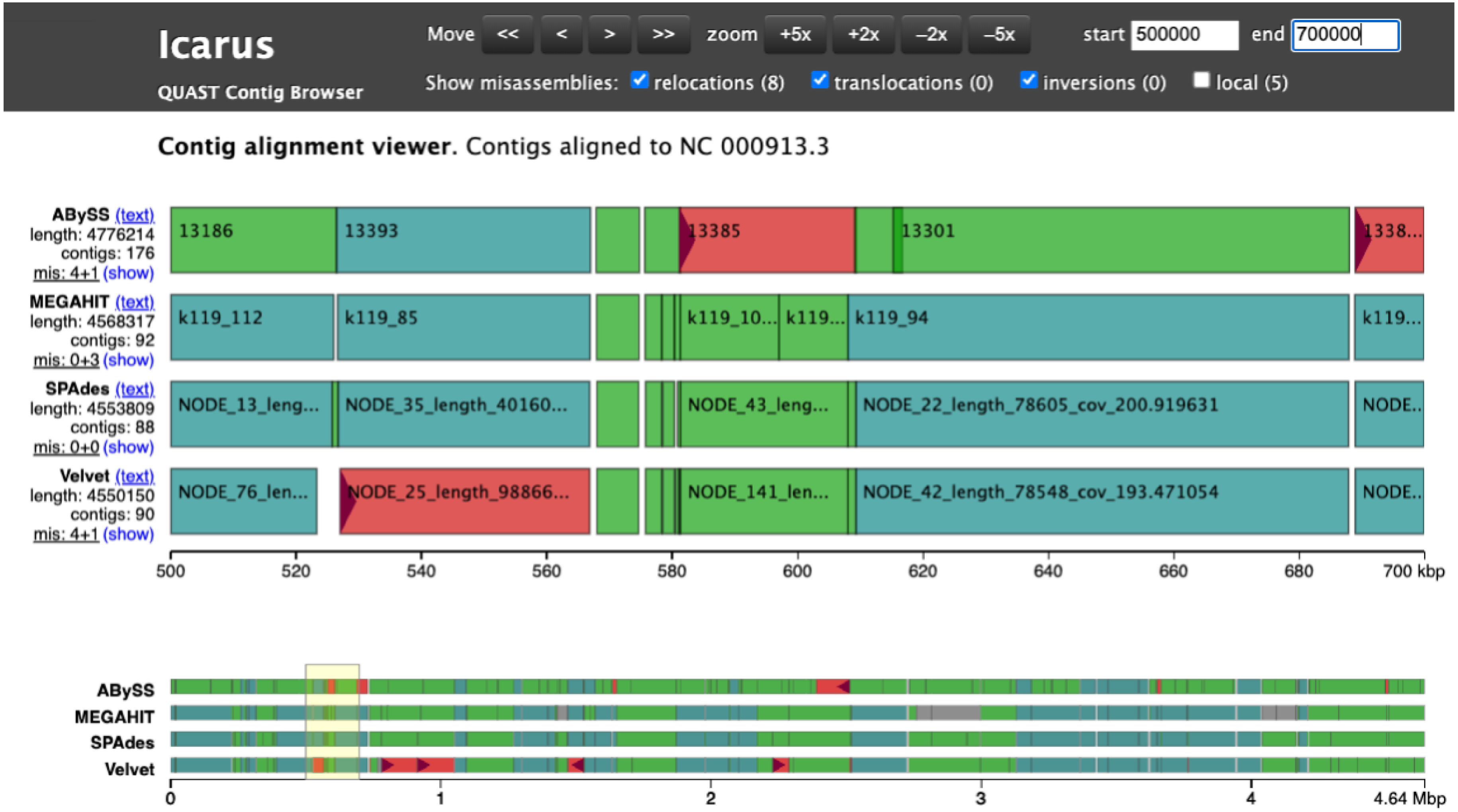

Supplementary Figure S4. WebQUAST Icarus viewer for *E. coli* assemblies aligned against the corresponding reference genome, *E.coli* str. K-12 substr. MG1655 (Use Case 2). The region between 0.5 Mbp and 0.7 Mbp is shown in the top detailed view panel and highlighted in yellow in the bottom overview panel. Correct contigs are green and aquamarine (if longer than 10 kbp and similar in at least three assemblies), and fragments of misassembled contigs are pink. The red triangle designates the misassembled side of a contig fragment. The full interactive report is available at https://www.ccb.uni-saarland.de/quast/sample_data_true_ref/icarus_viewers/alignment_viewer.html.

Aligned to "Ecoli_K12_W3110" | 4 646 332 bp | 1 fragment | 50.80% G+C

Worst Median Best ☑ Show heatmap

Alignment-based statistics	ABySS	MEGAHIT	SPAdes	Velvet
Genome fraction (%)	98.408	98.165	97.863	97.763
Duplication ratio	1.043	1	1	1
Largest alignment	191 920	235 933	264 217	264 944
Total aligned length	4 768 756	4 560 800	4 546 654	4 544 052
NG50	69 801	122 647	133 309	112 446
NG90	24 486	29 140	31 582	30 841
auNG	85 937	125 571	138 685	123 739
NA50	58 600	112 470	121 109	103 237
NA90	19 224	29 021	32 911	31 580
auNA	73 005	113 692	120 229	109 083
NGA50	64 383	112 470	121 109	103 237
NGA90	21 840	28 759	28 946	26 752
auNGA	75 071	111 856	117 927	106 874
LG50	21	14	12	14
LG90	62	41	39	42
LA50	25	15	14	16
LA90	75	45	43	47
LGA50	24	15	14	16
LGA90	70	47	46	50
**Misassemblies**				
# misassemblies	14	10	10	15
# relocations	14	10	10	15
# translocations	0	0	0	0
# inversions	0	0	0	0
# misassembled contigs	12	8	8	12
Misassembled contigs length	1 015 940	1 206 219	1 375 075	1 543 157
# local misassemblies	2	4	1	2
# scaffold gap ext. mis.	0	0	0	0
# scaffold gap loc. mis.	10	0	4	6
# unaligned mis. contigs	0	0	0	0
**Unaligned**				
# fully unaligned contigs	0	3	4	0
Fully unaligned length	0	2238	2936	0
# partially unaligned contigs	1	1	1	1
Partially unaligned length	6782	6759	6782	6818

(continued)

Worst Median Best ☑ Show heatmap

Per base quality	ABySS	MEGAHIT	SPAdes	Velvet
# mismatches per 100 kbp	2.14	2.87	1.17	3.52
# mismatches	102	131	53	160
# indels per 100 kbp	0.59	1.38	0.33	2
# indels	28	63	15	91
# indels (<= 5 bp)	11	38	8	28
# indels (> 5 bp)	17	25	7	63
Indels length	1469	3275	1095	4261
# N's per 100 kbp	24.59	0	17.55	94.19
# N's	1175	0	800	4288
**Statistics without reference**				
# contigs	176	95	92	90
# contigs (>= 0 bp)	4232	111	173	168
# contigs (>= 1000 bp)	149	82	80	79
# contigs (>= 5000 bp)	98	59	56	59
# contigs (>= 10000 bp)	85	55	52	56
# contigs (>= 25000 bp)	60	46	44	47
# contigs (>= 50000 bp)	36	31	29	32
Largest contig	248 481	235 933	285 196	264 944
Total length	4 777 853	4 571 292	4 557 363	4 552 266
Total length (>= 0 bp)	5 084 212	4 576 250	4 571 871	4 567 275
Total length (>= 1000 bp)	4 757 929	4 562 458	4 548 710	4 544 453
Total length (>= 5000 bp)	4 651 118	4 508 688	4 495 708	4 499 157
Total length (>= 10000 bp)	4 562 801	4 478 614	4 466 223	4 475 223
Total length (>= 25000 bp)	4 135 856	4 341 940	4 340 269	4 334 764
Total length (>= 50000 bp)	3 248 113	3 833 793	3 812 315	3 817 904
N50	67 616	122 647	133 309	125 360
N90	20 924	31 710	35 183	35 181
auN	83 572	127 633	141 393	126 296
L50	22	14	12	13
L90	68	39	37	39
GC (%)	50.71	50.74	50.74	50.73
**Similarity statistics**				
# similar correct contigs	16	29	30	28
# similar misassembled blocks	2	9	9	9

Short report

Supplementary Figure S5. Extended WebQUAST text report for *E. coli* assemblies evaluated against a close reference genome, *E.coli* str. K-12 substr. W3110 (Use Case 3). Unless otherwise noted, all statistics are based on contigs of size ≥ 500 bp (the default cut-off). The full interactive report is available at https://www.ccb.uni-saarland.de/quast/sample_data_close_ref/report.html.

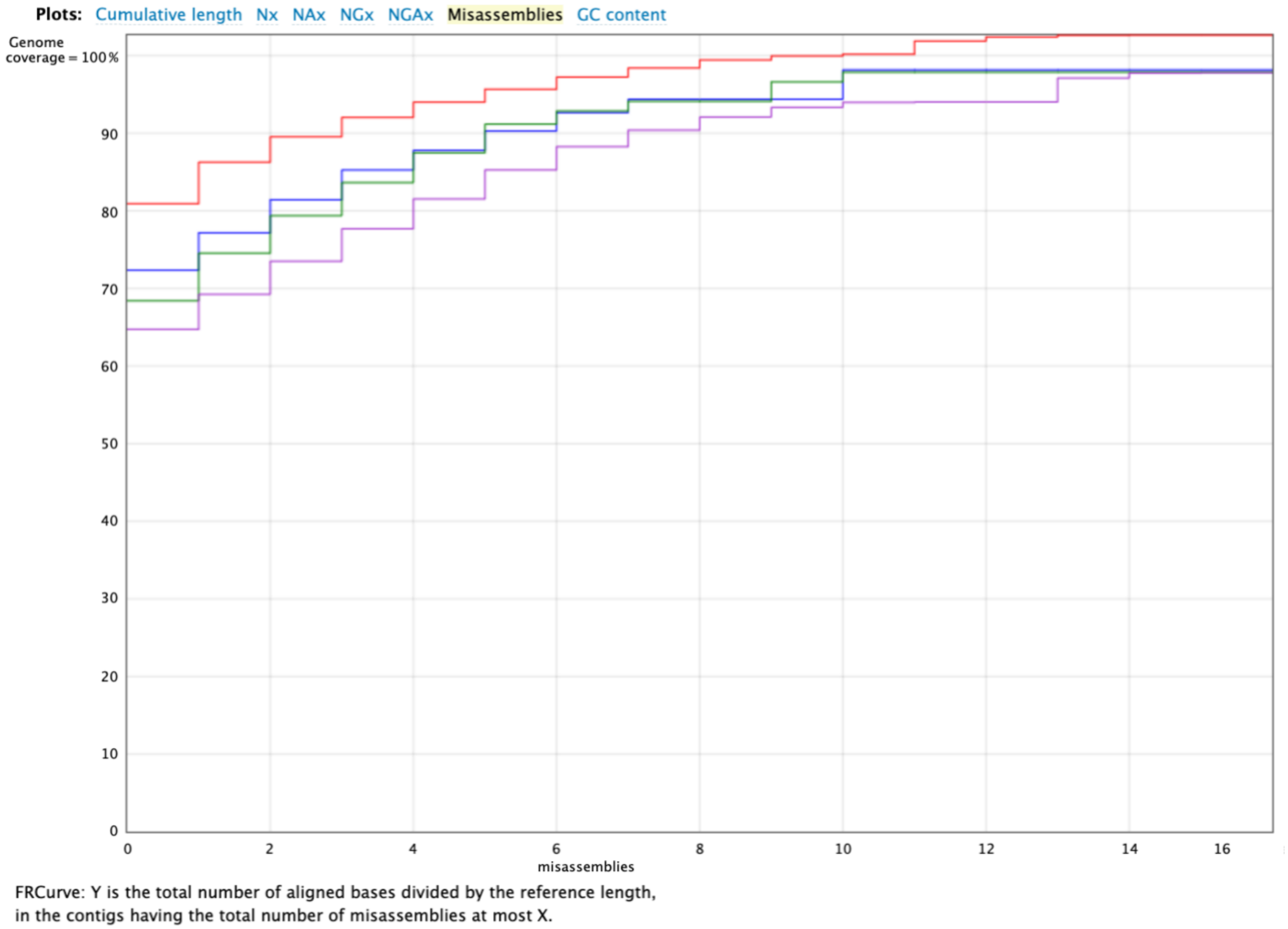

Supplementary Figure S6. Misassemblies feature-response curve for *E. coli* assemblies evaluated against a close reference genome, *E.coli* str. K-12 substr. W3110 (Use Case 3). The plot is based on contigs of size ≥ 500 bp (the default cut-off). The full interactive report is available at https://www.ccb.uni-saarland.de/quast/sample_data_close_ref/report.html.

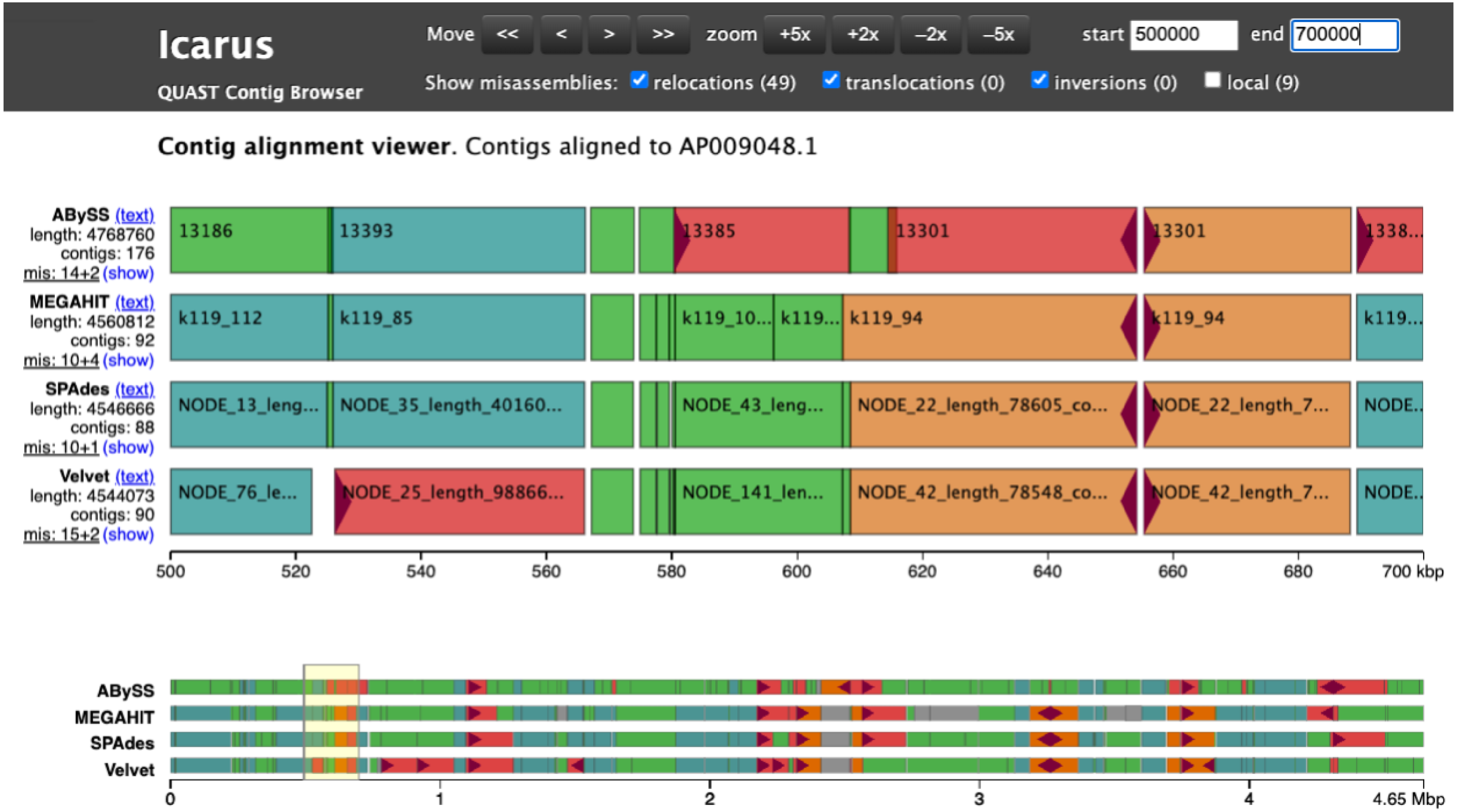

Supplementary Figure S7. WebQUAST Icarus viewer for *E. coli* assemblies evaluated against a close reference genome, *E.coli* str. K-12 substr. W3110 (Use Case 3). The region between 0.5 Mbp and 0.7 Mbp is shown in the top detailed view panel and highlighted in yellow in the bottom overview panel. Correct contigs are green and aquamarine (if longer than 10 kbp and similar in at least three assemblies), and fragments of misassembled contigs are pink and orange (if similar in at least three assemblies). The red triangle designates the misassembled side of a contig fragment. The full interactive report is available at https://www.ccb.uni-saarland.de/quast/sample_data_close_ref/icarus_viewers/alignment_viewer.html.

Running commands

Prerequisites

Installing all genome assemblers and auxiliary data processing tools via Bioconda

```
> conda install -c bioconda fastqc trimmomatic abyss megahit spades velvet

> fastqc --version
FastQC v0.12.1

> trimmomatic -version
0.39

> abyss-fac --version
abyss-fac (ABySS) 2.3.5

> megahit --version
MEGAHIT v1.2.9

> spades.py --version
SPAdes genome assembler v3.15.5

> velveth | head -n2
velveth - simple hashing program
Version 1.2.10
```

Raw sequencing data download

```
> wget http://ftp.sra.ebi.ac.uk/vol1/run/ERR008/ERR008613/200x100x100-081224_EAS20_0008_FC30TBBAAXX-6.tar.gz
> tar xzf 200x100x100-081224_EAS20_0008_FC30TBBAAXX-6.tar.gz
```

Reads quality control

```
> mkdir -p fastqc_report && fastqc -f fastq EAS20_8/s_6_1.fastq EAS20_8/s_6_2.fastq -o fastqc_report
```

Reads quality trimming

```
> trimmomatic PE EAS20_8/s_6_1.fastq EAS20_8/s_6_2.fastq -baseout trimmed_s_6 SLIDINGWINDOW:4:20
> mv trimmed_s_6_1P reads_1.fastq
> mv trimmed_s_6_2P reads_2.fastq
```

Genome assembly

ABySS (default parameters resulted in a very poor assembly, so we used parameters suggested in the GAGE-B study)

```
> abyss-pe -C abyss_out B=2G k=55 l=1 n=5 s=100 in='reads_1.fastq reads_2.fastq' name=ecoli
> cp abyss_out/ecoli-scaffolds.fa ABySS.fasta
```

MEGAHIT

```
> megahit -1 reads_1.fastq -2 reads_2.fastq -o megahit_out
> cp megahit_out/final.contigs.fa MEGAHIT.fasta
```

SPAdes

```
> spades.py -1 reads_1.fastq -2 reads_2.fastq -o spades_out
> cp spades_out/scaffolds.fasta SPAdes.fasta
```

Velvet (we used shuffleSequences_fastq.pl from the Velvet repository)

```
> wget https://raw.githubusercontent.com/dzerbino/velvet/master/contrib/MetaVelvet-v0.3.1/shuffleSequences_fastq.pl
> perl shuffleSequences_fastq.pl reads_1.fastq reads_2.fastq reads_interleaved.fastq
> velveth velvet_out  55 -fastq -shortPaired reads_interleaved.fastq
> velvetg velvet_out -exp_cov auto -cov_cutoff auto -ins_length 215 -scaffolding yes
> cp velvet_out/contigs.fa Velvet.fasta
```
